# Relationship between Resting Heart Rate, Blood Pressure and Pulse Pressure in Adolescents

**DOI:** 10.5935/abc.20170050

**Published:** 2017-05

**Authors:** Diego Giulliano Destro Christofaro, Juliano Casonatto, Luiz Carlos Marques Vanderlei, Gabriel Grizzo Cucato, Raphael Mendes Ritti Dias

**Affiliations:** 1 Universidade Estadual Paulista Júlio de Mesquita Filho (UNESP), Presidente Prudente, SP - Brazil; 2 Hospital Israelita Albert Einstein, São Paulo, SP - Brazil; 3 Universidade Norte do Paraná (UNOPAR), Londrina, PR - Brazil

**Keywords:** Heart Rate, Arterial Pressure, Rest, Adolescents

## Abstract

**Background:**

High resting heart rate is considered an important factor for increasing mortality chance in adults. However, it remains unclear whether the observed associations would remain after adjustment for confounders in adolescents.

**Objectives:**

To analyze the relationship between resting heart rate, blood pressure and pulse pressure in adolescents of both sexes.

**Methods:**

A cross-sectional study with 1231 adolescents (716 girls and 515 boys) aged 14-17 years. Heart rate, blood pressure and pulse pressure were evaluated using an oscillometric blood pressure device, validated for this population. Weight and height were measured with an electronic scale and a stadiometer, respectively, and waist circumference with a non-elastic tape. Multivariate analysis using linear regression investigated the relationship between resting heart rate and blood pressure and pulse pressure in boys and girls, controlling for general and abdominal obesity.

**Results:**

Higher resting heart rate values were observed in girls (80.1 ± 11.0 beats/min) compared to boys (75.9 ± 12.7 beats/min) (p ≤ 0.001). Resting heart rate was associated with systolic blood pressure in boys (Beta = 0.15 [0.04; 0.26]) and girls (Beta = 0.24 [0.16; 0.33]), with diastolic blood pressure in boys (Beta = 0.50 [0.37; 0.64]) and girls (Beta = 0.41 [0.30; 0.53]), and with pulse pressure in boys (Beta = -0.16 [-0.27; -0.04]).

**Conclusions:**

This study demonstrated a relationship between elevated resting heart rate and increased systolic and diastolic blood pressure in both sexes and pulse pressure in boys even after controlling for potential confounders, such as general and abdominal obesity.

## Introduction

High resting heart rate (RHR) has recently come to be considered an important factor for increasing the chance of mortality, and this relationship is independent of age, sex, lipid profile or blood pressure (BP) values in adults.^1^ High RHR tends to be associated with myocardial infarction,^2^ which could contribute to the likelihood of death from coronary heart disease in the future.

In previous studies,^3-5^ a relationship has been observed between elevated RHR and high BP in pediatric populations, which suggests that elevated RHR may be a marker of cardiovascular disease in childhood. This relationship could be mediated by other important cardiovascular risk factors such as obesity,^3,4^ which, through inflammatory substances, could increase RHR.^6^

Another factor that has been associated with high RHR is pulse pressure (PP), an important marker of vascular stiffness. A recent study^7^ has demonstrated a positive relationship between high RHR and PP in 227 healthy male African-American adolescents. However, as PP is affected by both BP levels and obesity status, it remains unclear whether the observed associations would remain after adjustment for these confounders.

Moreover the sex of adolescents should also be considered when analyzing these relationships. Rosa et al.^8^ have found that male adolescents had significantly higher PP values than female adolescents. Higher values of heart rate variability were observed in men compared to women.^9^ These findings show that hormonal and local fat deposition characteristics could influence RHR and other cardiovascular risk factors.

The purpose of this study was to analyze the association between RHR, BP and PP in adolescents of both sexes and to identify covariates of this association.

## Methods

This was a cross-sectional study carried out in the city of Londrina, Paraná state, situated in southern Brazil. A meeting was held with the Secretary of Education in the city of Londrina. As a result of that meeting, information was obtained on the six largest public schools in the downtown area, which were most likely to include students from different regions of the city (Northern, Southern, Eastern, Western and Central regions). Those schools were chosen for the study.

Londrina has approximately 17,000 enrolled students. To calculate the sample size, we used a correlation value of 0.18, an alpha error of 5%, a power of 80% and a design correction of 2.0 (as the sample was evaluated as clusters). Considering 20% losses, a minimum sample size of 624 students was required. The sample was composed of 1231 adolescents aged 14-17 years (515 boys and 716 girls). The inclusion criteria consisted in authorizing the consent form signed by parent/guardian allowing their adolescent to participate in the study, not be ingesting any medication or being in treatment related to problems of BP or heart rate. This study was approved by the ethics committee on human experimentation of the institution involved (process: 203/2010).

### Measurements

#### Anthropometry

Body weight was measured using an electronic scale (precision, 0.1 kg), with the subjects wearing light clothing, and height was measured with a wall-mounted stadiometer (precision, 0.1 cm). Body mass index (BMI) was calculated from the values of weight divided by height squared (kg/m^2^). Adolescents were classified as normal weight or overweight according to the cutoff points of the World Health Organization.^10^ All anthropometric measurements were performed by the same researcher, according to standardized techniques. Waist circumference was measured using an inextensible tape-measure, according to the values ​​recommended by Taylor et al.,^11^ considering the age and sex of the adolescents.

#### Resting heart rate

Resting heart rate and BP were evaluated using an oscillometric device (Omron HEM-742; Omron Corporation, Kyoto, Kansai, Japan), validated for adolescents.^12^ The participants sat silently in a room with their backs leaning against a chair and their arms flexed at an angle of 90 degrees. After 5 minutes of resting the first evaluation of RHR was performed, and after 2 minutes the second measurement was taken. The average of the two evaluations was used to determine RHR. These procedures were adopted according to the American Heart Society standards.^13^ Adolescents located in quartile 4 were classified as having high RHR and the others as having low RHR.

Systolic BP (SBP) and diastolic BP (DBP) were measured concomitantly with RHR. The mean value was used. To indicate the presence of high levels of BP, the 95th percentile of the National High Blood Pressure Education Program cutoffs was considered, adjusted by age and height percentile.^14^

Pulse pressure was calculated (difference between SBP and DBP).

### Statistical analysis

The sample characterization data were described as mean and standard deviation. As there is no specific cutoff point for RHR or PP in adolescents, we chose to classify the adolescents in the highest quartile as having a possible risk for those outcomes.

The number of adolescents classified according to the risk factors was analyzed by means of frequency and possible associations observed by using the Chi-square test. The multivariate analysis (Linear Regression) was applied and adjusted for sex and age (first model), overall fat determined by BMI (second model), and central fat (third model). The confidence interval was 95% and significance level, p < 0.05. All statistical analysis was performed using SPSS v.18.0.

## Results

Younger age had higher RHR values (14-15 years = 80.3 ± 12.0 beats/min; 16-17 years = 76.7 ± 12.4 beats/min [p = 0.001]). Higher RHR values were observed in girls (80.1 ± 11.0 beats/min) as compared to boys (75.9 ± 12.7 beats/min) (p ≤ 0.001). Elevated RHR was observed in 131 male adolescents (25.4%) and in 231 female adolescents (32.2%) (p = 0.011). There was no difference in BMI between boys and girls, but boys classified as having high RHR had higher BMI. The boys had higher waist circumference than girls, and the boys classified as having high RHR had higher waist circumference values. The sample characteristics are shown in Table 1.

**Table 1:** Characteristics of participants by sex and presence of resting heart rate (RHR)

	Boys (n = 515)		Girls (n = 716)	
	Normal RHR	High RHR	Normal RHR	High RHR
	Mean (SD)	Mean (SD)	Mean (SD)	Mean (SD)
Age (years)	15.5 (1.1)	15.2 (1.1)	15.7 (0.9)	15. 3 (1.0)
Weight (kg)	64.1 (12.7)	66.5 (16.8)^a,b^	56.9 (11.5)	56.5 (11.3)
Stature (cm)	173.2 (8.3)	171.1 (9.5)^a^	162.4 (6.7)	162.1 (6.6)
BMI (kg/m^2^)	21.2 (3.4)	22.6 (5.2) ^b^	21.5 (3.7)	21.5 (3.9)
WC (cm)	74.1 (8.8)	77.9 (12.2)^a,b^	70.5(8.8)	70.5 (8.4)
SBP (mm Hg)	119.7 (10.7)	121.9 (12.4) ^a,b^	110.0 (3.7)	113.2 (3.9) ^b^
DBP (mm Hg)	63.4 (7.5)	67.5 (8.4) ^b^	64.2 (7.4)	66.8 (7.8) ^b^
PP	56.2 (9.5)	54.4 (9.9) ^a^	45.8 (7.0)	46.4 (7.4)

Difference between groups evaluated by two-way ANOVA;

a p < 0.05 for the difference between boys and girls;

b p < 0.05 for the difference between high RHR and normal RHR; RHR: resting heart rate; SD: standard deviation; kg: kilograms; cm: centimeters; BMI: body mass index; WC: waist circumference; SBP: systolic blood pressure; DBP: diastolic blood pressure; PP: pulse pressure.

A low correlation was observed between BMI and RHR (r = 0.06). Adolescents with higher RHR had a higher prevalence of high BP (36.4% vs. 28.4%; p = 0.050). There was no significant difference in RHR in adolescents when stratifying for nutritional status (p = 0.174) or PP values (p = 0.158). Table 2 shows the correlation between RHR and SBP, DBP and PP. Considering the relationship between heart rate and SBP and DBP, for each increased heartbeat there is a 0.090-mmHg increase in SBP of boys and a 0.063 mmHg increase in SBP of girls. Regarding DBP, the increase is 0.179 mmHg in boys and 0.161 mmHg in girls.

**Table 2:** Correlation between resting heart rate and systolic and diastolic blood pressure and pulse pressure

Independent Variable	Pearson Correlation (dependent variable: RHR)	
	r	p-value
**Male**		
SBP	0.10	≤ 0.001
DBP	0.23	≤ 0.001
PP	-0.10	0.016
**Female**		
SBP	0.19	≤ 0.001
DBP	0.24	≤ 0.001
PP	-0.01	0.658

RHR: resting heart rate, SBP: systolic blood pressure; DBP: diastolic blood pressure; PP: pulse pressure.

The linear regression models adjusted for sex, age, overall fat and central obesity indicated that RHR was associated with SBP (p ≤ 0.05) and DBP (p ≤ 0.05) for both sexes, and with PP (p = 0.05) only for boys (Tables 3 and 4).

**Table 3:** Relationship between resting heart rate and systolic and diastolic blood pressure and pulse pressure in boys

	Resting Heart Rate								
	Adjusted: age			Adjusted: age and BMI			Adjusted: age, BMI and WC		
	Beta	p-value	95%CI	Beta	p-value	95%CI	Beta	p-value	95%CI
SBP	0.17	≤ 0.001	0.07;0.27	0.15	0.003	0.05;0.25	0.15	0.014	0.04;0.26
DBP	0.51	≤ 0.001	0.38;0.65	0.50	≤ 0.001	0.37;0.63	0.50	≤ 0.001	0.37;0.64
PP	-0.12	0.035	-0.02; -0.09	-0.15	0.011	-0.26; -0.03	-0.16	0.008	-0.27; -0.04

BMI: body mass index; WC: waist circumference; CI: confidence interval; SBP: systolic blood pressure; DBP: diastolic blood pressure; PP: pulse pressure.

**Table 4:** Relationship between resting heart rate and systolic and diastolic blood pressure and pulse pressure in girls

	Resting Heart Rate								
	Adjusted: age			Adjusted: age and BMI			Adjusted: age, BMI and WC		
	Beta	p-value	95%CI	Beta	p-value	95%CI	Beta	p-value	95%CI
SBP	0.23	≤0.001	0.15;0.32	0.24	≤0.001	0.15;0.33	0.24	≤0.001	0.16;0.33
DBP	0.40	≤0.001	0.29;0.51	0.40	≤0.001	0.30;0.51	0.41	≤0.001	0.30;0.53
PP	-0.03	0.534	-0.15;0.08	-0.04	0.488	-0.17;0.08	-0.05	0.438	-0.17;-0.07

BMI: body mass index; WC: waist circumference; CI: confidence interval; SBP: systolic blood pressure; DBP: diastolic blood pressure; PP: pulse pressure.

## Discussion

This study's results indicate that adolescents with higher RHR values have higher SBP and DBP values. High RHR was associated with higher SBP and DBP in males and females, and was inversely related to PP only in male adolescents.

This study's results are in agreement with those reported by Liu et al.,^15^ who, assessing more than 8,000 individuals from different countries, aged 48-56 years, have found that high RHR associated with high BP values. Similar findings were observed by Kwok et al.,^4^ who evaluated the relationship between high values of RHR and of BP in young Chinese individuals. Corroborating our study, Fernandes et al.^3^ have demonstrated similar relationships in 356 male adolescents. Even after controlling for different variables in the cited studies, high BP values were associated with high RHR. One reason is that heart rate and SBP and DBP are modulated by the central nervous system through sympathetic activity in the heart and vessels.^16^

Pulse pressure is an indicator of arterial stiffness of different arteries and vessels and has been related to arteriosclerotic processes.^17^ This study's results indicated that RHR was correlated with PP only in male adolescents, which is in agreement with those reported by Song et al.^18^ in a representative sample of Korean adolescents. One possible mechanism is the fact that PP is an indicator of arterial stiffness, which contributes to heart rate changes mediated by increased velocity of the pulse wave from the aorta to the peripheral vessels; in this case, the higher the speed of the anterograde wave, the higher the successive retrograde wave, causing cardiovascular overload.^19^

Another factor to be considered in this study is the relationship between obesity and increased heart rate. In a study of over 30,000 young people, Babba et al.^20^ have observed that RHR was strongly associated with obesity, added to which, average values of heart rate increased according to the degree of obesity. Adipose tissue releases a variety of substances, including adiponectin, which could contribute to changes in the sympathetic nervous system and decreased parasympathetic nervous system,^21^ increasing RHR values. Therefore, obesity is an important confounding factor to be considered in the analyses. The correlation between RHR and PP remained significant after adjustment for age, age + BMI, and age + BMI + waist circumference in males, suggesting an independent relationship between PP and RHR. A previous study^16^ has found no correlation between total fat mass and cardiovascular variables (systolic volume, SBP and RHR) in male adolescents, unlike abdominal fat. On the other hand, in female adolescents, the relationships are absolutely inverse, the cardiovascular variables (systolic volume, SBP and RHR) are directly related to total fat mass and unrelated to waist circumference.^18^

Concerning sex, the present study demonstrated associations between high RHR, SBP and DBP in both sexes; however, when considering RHR and PP, this relationship was observed only in male adolescents. Pulse pressure differences in boys and girls were observed by Rosa et al.^8^ after evaluating 456 adolescents, with higher PP values found in male adolescents. One possible difference to be considered is the age range of students in the current study (14-17 years), menstruation is a period of intense activity in girls and may alter the autonomic system due to intense hormonal production,^22^ leading to differences compared to boys. The location of body fat could also be related to higher RHR values. This has been clearly observed by Song et al.,^18^ who have found a direct relationship between cardiovascular variables (systolic volume, SBP and RHR) and waist circumference in male adolescents.

Regarding practical applications, it should be emphasized that such assessments need to be conducted from the earliest ages, aimed at preventing future cardiovascular diseases. Oscillometric devices have been used in several studies,^4,23^ and in addition to their ease of use, they typically provide values of BP and heart rate, which could be assessed in the school environment, enabling the early control of those risk factors.

One limitation of this study was its cross-sectional design, which does not allow assessment of the possible cause and effect relationships. Heart rate and BP were evaluated on the same day, which is known to cause overestimation of values.^24^ Another limiting aspect is the fact that PP was measured indirectly from the SBP and DBP values, and not from the pulse wave speed.

## Conclusions

In conclusion, male adolescents with higher RHR had higher SBP, DBP and PP values. In female adolescents, RHR was associated with SBP and DBP, but not with PP. Thus, health promotion activities should be encouraged in young populations, because cardiovascular risk factors interact. However, inherent characteristics of sex must be considered.
